# Phenotyping the quality of complex medium components by simple online-monitored shake flask experiments

**DOI:** 10.1186/s12934-014-0149-5

**Published:** 2014-11-07

**Authors:** Sylvia Diederichs, Anna Korona, Antje Staaden, Wolfgang Kroutil, Kohsuke Honda, Hisao Ohtake, Jochen Büchs

**Affiliations:** AVT – Biochemical Engineering, RWTH Aachen University, Worringerweg 1, D-52074 Aachen, Germany; Department of Chemistry, Organic and Bioorganic Chemistry, NAWI Graz, University of Graz, Heinrichstrasse 28, A-8010 Graz, Austria; Department of Biotechnology, Graduate School of Engineering, Osaka University, 2-1 Yamadaoka, Suita, Osaka 565-0871 Japan

**Keywords:** RAMOS, OTR, Yeast extract, Complex medium, *E. coli*, Auto-induction, Screening, Lactose, PAT

## Abstract

**Background:**

Media containing yeast extracts and other complex raw materials are widely used for the cultivation of microorganisms. However, variations in the specific nutrient composition can occur, due to differences in the complex raw material ingredients and in the production of these components. These lot-to-lot variations can affect growth rate, product yield and product quality in laboratory investigations and biopharmaceutical production processes. In the FDA’s Process Analytical Technology (PAT) initiative, the control and assessment of the quality of critical raw materials is one key aspect to maintain product quality and consistency. In this study, the Respiration Activity Monitoring System (RAMOS) was used to evaluate the impact of different yeast extracts and commercial complex auto-induction medium lots on metabolic activity and product yield of four recombinant *Escherichia coli* variants encoding different enzymes.

**Results:**

Under non-induced conditions, the oxygen transfer rate (OTR) of *E. coli* was not affected by a variation of the supplemented yeast extract lot. The comparison of *E. coli* cultivations under induced conditions exhibited tremendous differences in OTR profiles and volumetric activity for all investigated yeast extract lots of different suppliers as well as lots of the same supplier independent of the *E. coli* variant. Cultivation in the commercial auto-induction medium lots revealed the same reproducible variations. In cultivations with parallel offline analysis, the highest volumetric activity was found at different cultivation times. Only by online monitoring of the cultures, a distinct cultivation phase (e.g. glycerol depletion) could be detected and chosen for comparable and reproducible offline analysis of the yield of functional product.

**Conclusions:**

This work proves that cultivations conducted in complex media may be prone to significant variation in final product quality and quantity if the quality of the raw material for medium preparation is not thoroughly checked. In this study, the RAMOS technique enabled a reliable and reproducible screening and phenotyping of complex raw material lots by online measurement of the respiration activity. Consequently, complex raw material lots can efficiently be assessed if the distinct effects on culture behavior and final product quality and quantity are visualized.

**Electronic supplementary material:**

The online version of this article (doi:10.1186/s12934-014-0149-5) contains supplementary material, which is available to authorized users.

## Background

A biopharmaceutical or biotechnological production process consists of many critical elements that can influence the consistency of process performance and final product quality. The FDA’s Process Analytical Technology (PAT) initiative [1] addresses problems of inconsistent production outcome by real-time analysis and control of critical production steps. The aim of the PAT initiative is to ensure a reproducible product quality by enhancing the understanding and control of manufacturing processes. The control and assessment of the quality of critical raw materials that are used for the preparation of cultivation media is one key aspect to maintain or even improve product quantity and quality [2-5].

Common media for the cultivation of bacteria, yeast, fungi, and other organisms contain complex medium components [6]. Contrary to synthetic media which are composed of pure chemicals in known concentrations [7], complex media include chemically undefined components of natural origin like yeast extract, meat extract, peptone, casein hydrolysate, carcass meal, or plant seed flour. Among those medium components, yeast extract is one of the most widely used medium supplements. It consists of a mixture of carbohydrates, amino acids, peptides, vitamins, and trace elements [6]. The composition of yeast extracts can exhibit dramatic lot-to-lot variations due to differences in yeast strains, yeast production processes, methods for autolysis, or downstream purification [6]. Hence, a change of the yeast extract lot employed for the preparation of growth medium might have tremendous effects on growth rate and product yield, as already described in several studies for different organisms [6,8-12]. Fu et al. demonstrated that the yield of a fusion protein (thioredoxin-human parathyroid hormone) can vary in *E. coli* cultivations without addition of an exogenous inducer when the strain was cultivated in complex medium supplemented with yeast extracts of three different manufacturers [8]. Sorensen et al. recently reported an influence of different yeast extracts on the production of several secondary metabolites in species of the fungi *Fusarium* [12]. Zhang et al. tested 40 yeast extracts in fermentations of recombinant *Saccharomyces cerevisiae* and found adenine, trehalose, and lactate affecting growth and product formation [6]. When cultivating a *Penicillium* strain, Baracat-Pereira et al. reported that possible precursors or inducers of cAMP in yeast extract could enhance the induction of protein expression [13]. Based on these findings, the identification of yeast extract ingredients promoting growth and production rate is of special interest to achieve a consistent fermentation performance. However, since the regulation of the cellular metabolism is very complex, it might be difficult to determine all ingredients that affect cell growth and productivity. In addition, the same expression host might require a differed nutrient composition in the medium when producing different recombinant proteins [14,15]. Therefore, even a detailed evaluation of many ingredients in yeast extract might not automatically allow reliable conclusions on yield and quality of a specific product.

Rather than focusing on a detailed analysis of the various ingredients of different yeast extracts, it could, thus, be advantageous to develop a simple and rapid screening system to evaluate the culture performance concerning growth and product formation. This approach would enable a fast, efficient and detailed investigation of the effect of lot-to-lot variations based on the production host in hand. However, none of the published methods and systems meets all criteria for an efficient screening and phenotyping of yeast extracts, such as determination of culture growth, specific product yield, and easy implementation. Potvin et al. assessed a microtiter plate based automated turbidimetry system to screen various yeast extracts for their growth promoting ability of *Lactobacillus* cultures [11]. This system enabled parallel evaluation of yeast extracts in terms of culture growth but did not determine product yield. For cell culture processes, Iding et al. investigated a bioreactor-based test system to display the effects of different yeast extract lots on the oxygen-dependent cell metabolism of a mouse-mouse hybridoma cell line as measure of cell activity [9]. The suitability of this system for screening is limited due to a very complex bioreactor design and a lack of parallel cultivations. Kasprow et al. analyzed yeast extracts by near-infrared spectroscopy and correlated the absorption spectra with biomass production and product yield in large-scale fermentation [10]. However, the transfer of the modelled data to cultivations at 2-L scale was not successful for biomass yield. Li et al. recently published a method to rapidly screen for consistency of yeast extract lots using surface-enhanced Raman scattering and fluorescence spectroscopy [16]. Yeast extracts could easily be distinguished by this method but a correlation of the results with the ability to promote growth or product formation of a certain host organism was not accomplished.

A promising tool for comprehensive, parallel studies of the influence of lot-to-lot variations is the Respiration Activity Monitoring System (RAMOS) established by Anderlei and Büchs [17] and by Anderlei et al. [18]. It enables an efficient screening and phenotyping of yeast extracts in parallel shake flask experiments. With this technique, the oxygen transfer rate (OTR) as function of time can be determined online in a non-invasive manner as parameter for growth and metabolic activity to control and assess the quality of different yeast extract lots. Previous studies already demonstrated the wide range of possible applications of this technique like optimization of culture conditions [19-23], characterization of recombinant organisms [14,24], detection of toxic effects of materials [25,26], and visualization of cultivation phenomena like secondary substrate limitation, diauxic growth or oxygen limitation [17,20,22,23].

**Figure 1:**
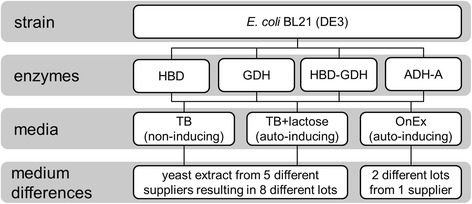
**Overview of conducted experiments.** Cultivations were performed with four *E. coli* BL21 (DE3) variants carrying the genes for 3-hydroxybutyryl-CoA dehydrogenase (*E. coli* HBD), glucose 1-dehydrogenase (*E. coli* GDH), 3-hydroxybutyryl-CoA dehydrogenase and glucose 1-dehydrogense (*E. coli* HBD-GDH), and alcohol dehydrogenase A (*E. coli* ADH-A) in three different complex media. The non-inducing TB medium and auto-inducing TB+lactose medium were prepared with yeast extracts from five different suppliers and, in total, eight different lots which are listed in Table 1. Additionally, cultivations were performed with two different lots of the commercially available Overnight Express (OnEx) auto-induction medium

*Escherichia coli* was chosen as model organism because it is the predominant bacterial expression system for the production of heterologous proteins [27-30] and it is frequently cultivated in complex media. Hence, the objective of this study was the employment of the RAMOS device for the evaluation of the impact of in total 8 different yeast extracts (from different suppliers and in different lots) on metabolic activity and recombinant protein yield of different recombinant *E. coli* variants (Figure 1). In addition, two lots of a commercial complex medium (Overnight Express (OnEx) auto-induction medium, Novagen, Merck, Germany) were analyzed to further prove the impact of lot-to-lot variation on metabolic activity during protein production.

Protein production was investigated in auto-induction media with lactose as natural inducer to facilitate the comparison of cultures only differing in yeast extract or commercial complex medium lot. In the auto-induction medium, initial biomass production and the subsequent heterologous protein expression are separated due to diauxic growth on the supplemented carbon sources glucose, lactose, and glycerol [31-33]. *E. coli* starts to grow on glucose as preferred carbon source repressing the uptake of lactose and glycerol. After depletion of glucose, lactose and glycerol are consumed in parallel accompanied by induction of recombinant protein production. Therefore, auto-induction media with the same mixture of carbon sources lead to parallel induction of cultures at the same biomass concentration without addition of an exogenous inducer.

## Results and discussion

### Cultivation under non-induced conditions

**Figure 2:**
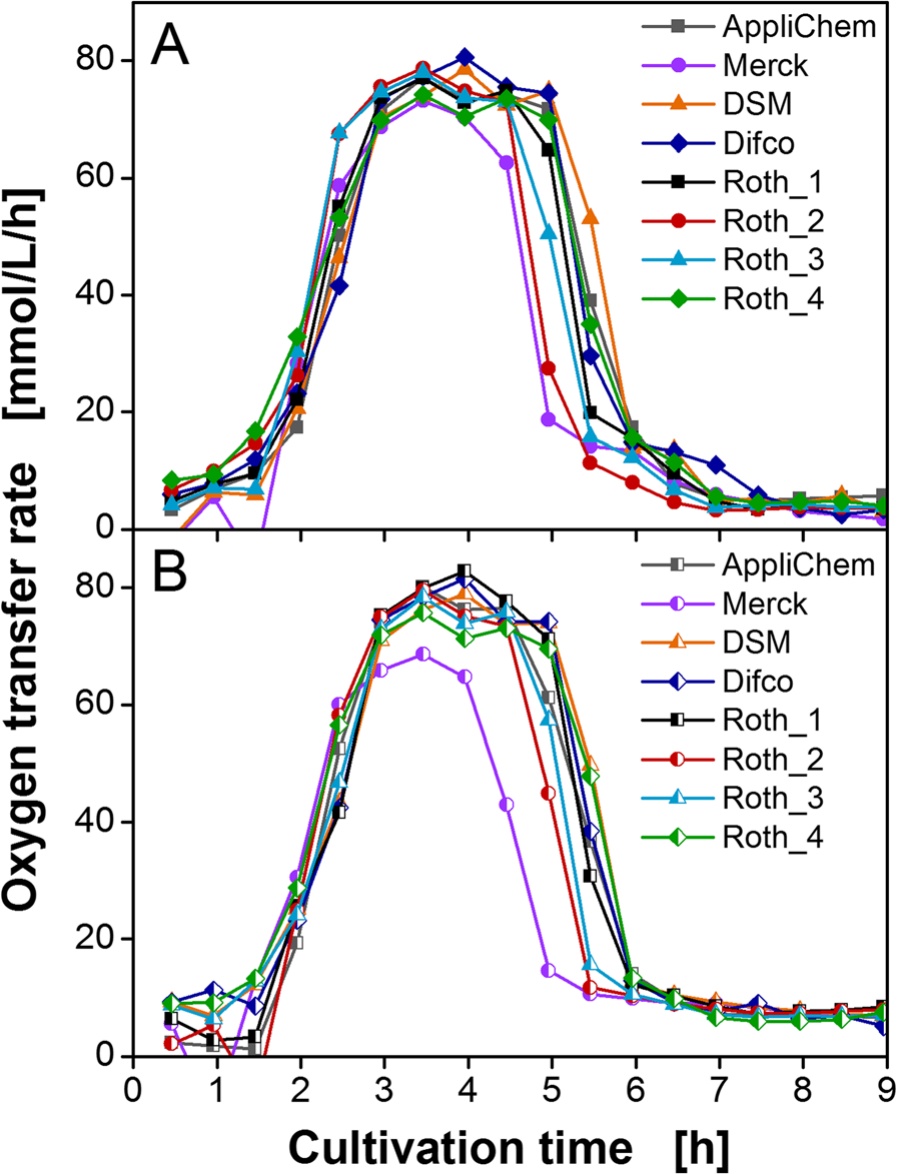
**Cultivation of** ***E. coli*** **under non-induced conditions.** Oxygen transfer rate during cultivation of two *E. coli* BL21 (DE3) variants carrying the genes for **(A)** 3-hydroxybutyryl-CoA dehydrogenase (*E. coli* HBD) and **(B)** glucose 1-dehydrogenase (*E. coli* GDH) in non-inducing TB medium with different yeast extracts as specified in Table 1. Conditions: 250-mL flask, filling volume 10 mL, shaking frequency 350 rpm, shaking diameter 50 mm, and 37°C.

Cultivations were conducted in the RAMOS device under non-inducing conditions in TB medium to evaluate the influence of the eight yeast extract lots (Table 1) on growth behavior of *E. coli*. Figure 2 shows the OTR of two recombinant *E. coli* variants harboring the gene for the 3-hydroxybutyryl-CoA dehydrogenase from *Thermus thermophilus* (*E. coli* HBD, Figure 2A) and for the glucose 1-dehyrogenase from *Sulfolobus solfataricus* (*E. coli* GDH, Figure 2B). The experiments were performed in 250-mL RAMOS flasks containing 10 mL TB medium with different yeast extract lots. The shapes of all depicted OTR curves as function of time were similar reaching a maximum OTR between 70 and 80 mmol/L/h after an exponential increase. After a cultivation time of 5 to 7.5 h, the OTR dropped to about 5 to 10 mmol/L/h. At this time, all available carbon sources were depleted and no further respiration was possible. Cultivations in TB medium prepared with Merck yeast extract reached the lowest maximum OTR and the shortest duration of respiration activity for both investigated strains. Since the TB medium differed only in yeast extract lot, Merck yeast extract exhibited inferior growth properties as compared to the other lots tested.

**Table 1:** **Yeast extracts and OnEx auto-induction medium lots analyzed in this work**

**Supplier**	**Product name**	**Lot-Nr.**	**Abbreviation**
AppliChem	Yeast extract BioChemica	20001783	AppliChem
Merck	Yeast extract for biotechnology, Fermtech®	VM434226218	Merck
DSM Food Specialties	Gistex LS (Ferm) Powder	GLFaU-0113	DSM
BD Bioscience (formerly Difco Laboratories)	Bacto yeast extract	132387JC	Difco
Roth	Yeast extract powder, for bacteriology	102184042	Roth_1
Roth	Yeast extract powder, for bacteriology	273201081	Roth_2
Roth	Yeast extract powder, for bacteriology	191169829	Roth_3
Roth	Yeast extract powder, for bacteriology	180156693	Roth_4
Novagen (Merck)	Overnight Express Instant TB medium	7190	OnEx_1
Novagen (Merck)	Overnight Express Instant TB medium	D00130327	OnEx_2

Comparison of the OTR curves in Figure 2 to published *E. coli* cultivations [14,22,24] revealed a typical respiration behavior of *E. coli* in TB medium independent of the yeast extract. Kunze et al. reached similar maximum OTRs and comparable durations of respiration activity under the same cultivation conditions [24]. This fact indicates that all yeast extracts covered the demand for growth of *E. coli* until depletion of the supplemented carbon source.

These findings are in contrast to studies on growth behavior of other organisms in medium supplemented with different lots of yeast extract. Iding et al. detected differences in growth behavior of a mouse-mouse hybridoma cell line depending on the yeast extract lot used [9] and Potvin et al. observed the same effect in cultivation of *Lactobacillus plantarum* with maximum biomass levels varying by 40% [11]. Consequently, *E. coli* growth appears to be less affected by variations in yeast extract composition than that of the organisms mentioned above. Hence, all of the tested yeast extract lots were applicable for biomass production of *E. coli* at non-inducing conditions.

### Reproducibility of RAMOS cultivations

**Figure 3:**
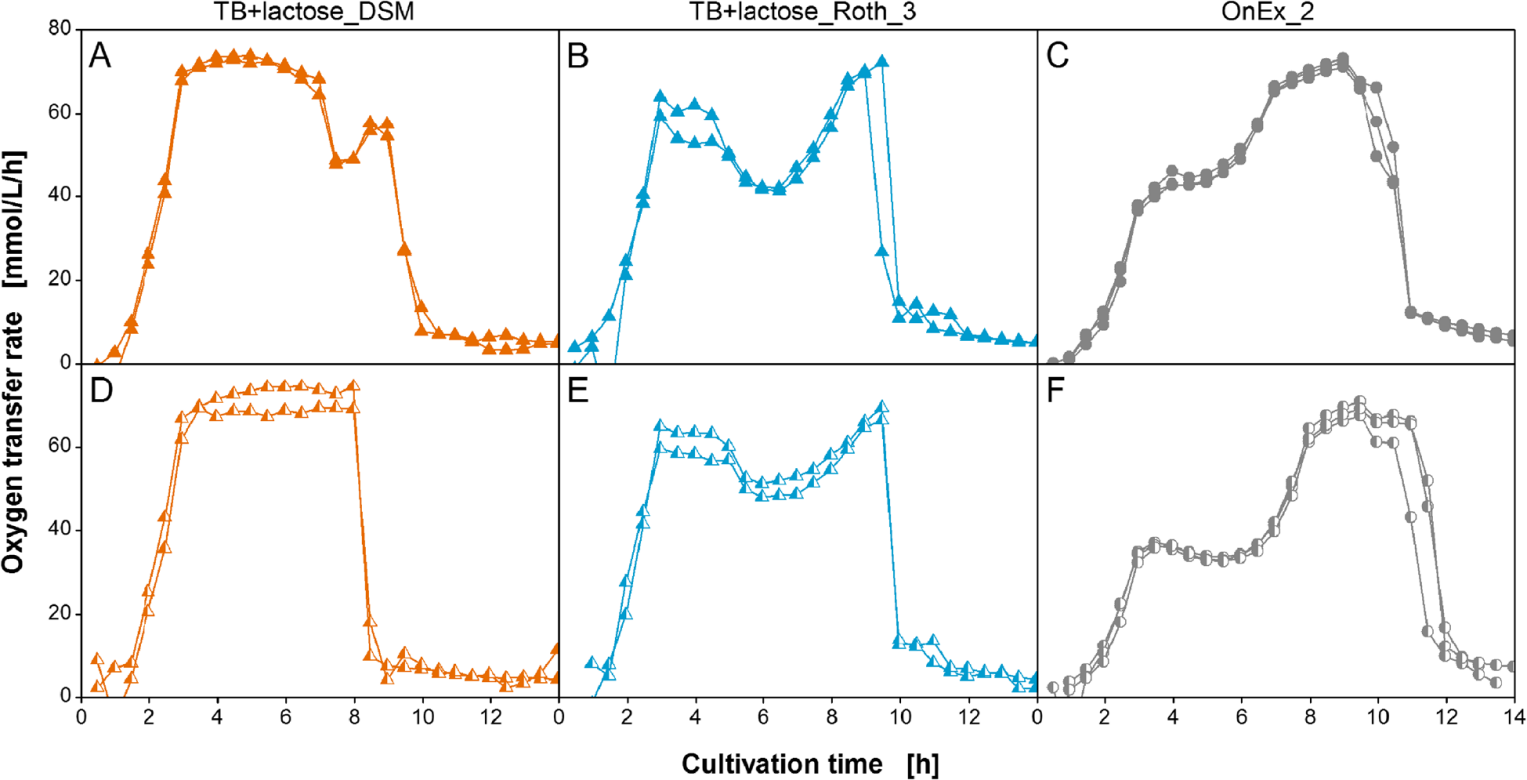
**Reproducibility of RAMOS cultivations in auto-induction media.** Oxygen transfer rate during independent cultivations of *E. coli* BL21 (DE3) variants expressing **(A-C)** 3-hydroxybutyryl-CoA dehydrogenase (*E. coli* HBD) and **(D-F)** glucose 1-dehydrogenase (*E. coli* GDH) in two auto-inducing TB+lactose media with different yeast extracts (DSM and Roth_3) and one Overnight Express auto-induction medium (OnEx_2) as specified in Table 1. Conditions: 250-mL flask, filling volume 10 mL, shaking frequency 350 rpm, shaking diameter 50 mm, and 37°C.

The following section focuses on the reproducibility of RAMOS cultivations in auto-induction media. This issue needs to be addressed to demonstrate that the RAMOS device is able to generate reliable and reproducible results for cultivations conducted independently and that all observed variations in respiration activity in the following experiments have to be attributed to changes in the chemical composition of the applied complex media. For this purpose, the results of independent cultivations in three different media are presented in Figure 3.

The independent cultivations revealed an excellent reproducibility since all cases exhibited the same general course of the OTR curves as function of time. In Figure 3A and C, no differences between the curves could be observed. Others showed only slight variations of less than 5 mmol/L/h in maximum OTR (Figure 3B, D, and E) or in the duration of respiration activity until the final decline of OTR (Figure 3F). Hence, *E. coli* cultivations in media supplemented with the same complex component lots provide specific and reproducible results. These results lead to the conclusion that the RAMOS device is suitable for comparing respiration activity of *E. coli* in auto-induction media between independent experiments.

### Cultivation under induced conditions using auto-induction media

*E. coli* is the main host for production of recombinant proteins in laboratory investigations. Therefore, the effect of changing lots of complex components on the efficiency of protein expression is of special interest. The respiration behavior and product formation of different *E. coli* BL21 (DE3) variants in various auto-induction media were compared. The variants were either cultivated in the self-made TB+lactose medium prepared with different yeast extract lots or in two different lots of the commercial Overnight Express (OnEx) auto-induction medium (Table 1). Both media are based on non-inducing TB medium with glycerol as main carbon source supplemented with glucose and lactose. To ensure comparability, the carbon source concentrations in TB+lactose medium were adjusted to the concentrations in OnEx_1 medium previously determined by Kunze et al. via HPLC analysis [24].

**Figure 4:**
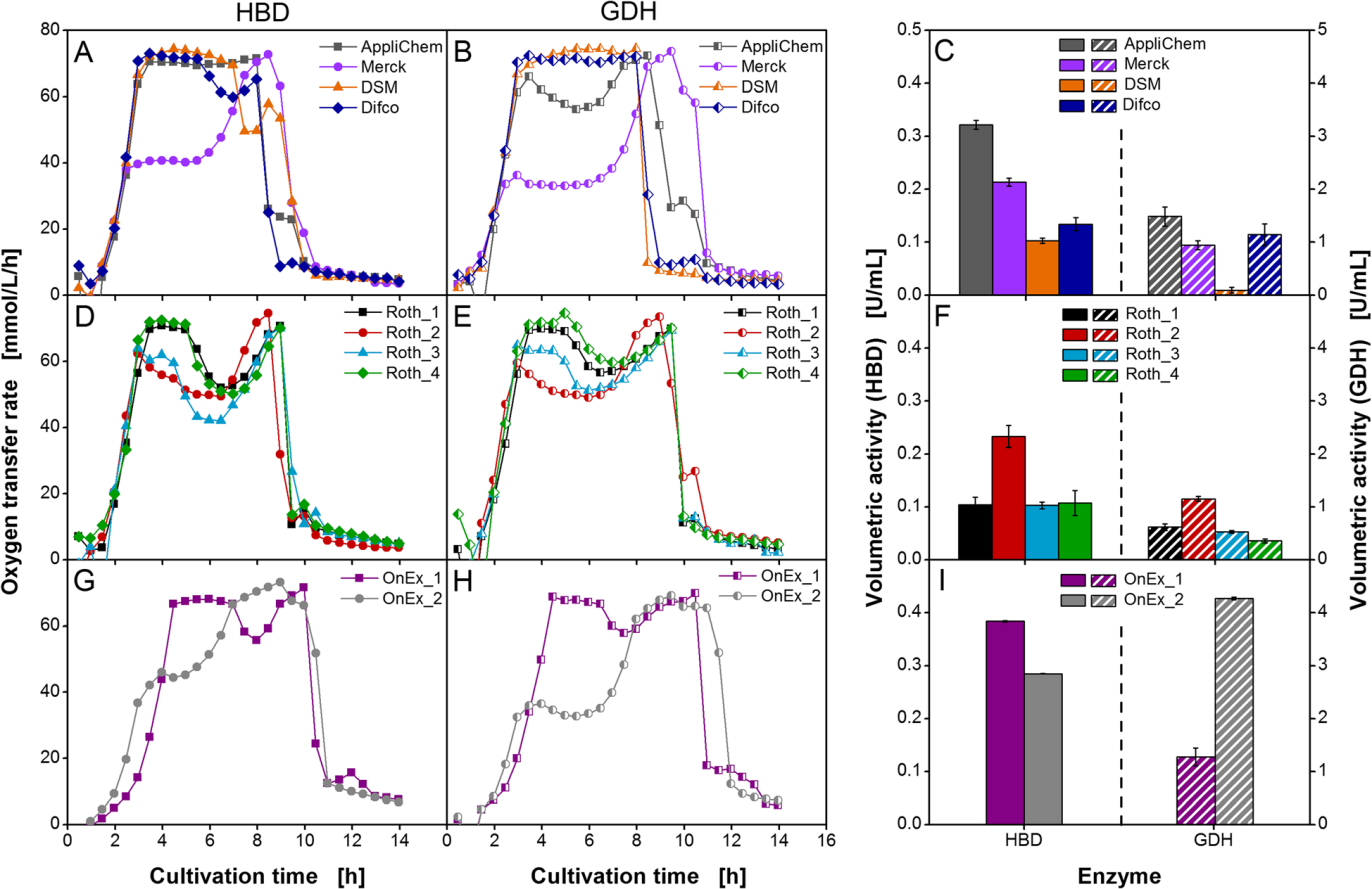
**Cultivation of** ***E. coli*** **under induced conditions using auto-induction media.** Oxygen transfer rate during cultivation of two *E. coli* BL21 (DE3) variants expressing 3-hydroxybutyryl-CoA dehydrogenase (*E. coli* HBD) and glucose 1-dehydrogenase (*E. coli* GDH) in **(A-B, D-E)** self-made auto-inducing TB+lactose medium with various yeast extracts as listed in Table 1 and **(G-H)** two different lots of commercial Overnight Express (OnEx) auto-induction medium. The respiration behavior is categorized into two patterns. Pattern I (Merck, Roth_2, and OnEx_2) is characterized by an initial increase in OTR to a plateau of 35 to 60 mmol/L/h and a second increase to 70 mmol/L/h, whereas the highest OTR is reached immediately in pattern II (AppliChem, DSM, Difco, Roth_1, Roth_4, and OnEx_1). Conditions: 250-mL flask, filling volume 10 mL, shaking frequency 350 rpm, shaking diameter 50 mm, and 37°C. **(C, F, I)** The volumetric enzyme activity of expressed HBD and GDH was measured in triplicates for all cultivations after 14 h.

In Figure 4, the OTR during cultivation of *E. coli* HBD (first column) and *E. coli* GDH (second column) and the volumetric activities of HBD and GDH at the end of the cultivation (third column) are presented for all tested auto-induction media. In contrast to the very similar respiration activity in non-inducing TB medium, the OTR exhibited tremendous differences in the shape of the curves in auto-inducing TB+lactose medium. The variation in OTR clearly indicated that the auto-inducing media affected the metabolic activity of the host cells depending on yeast extract lots and OnEx medium lots used to prepare the media. Interestingly, the general shapes of the OTR curves as function of time of the two *E. coli* variants were comparable in the same lots. The different shapes of respiration behavior in auto-induction media compared to cultivation under non-induced conditions (Figure 2) can be assigned to the different availability of carbon sources and to metabolic burden due to overproduction of plasmid-encoded recombinant proteins [34-37]. Different shapes of respiration behavior in the applied auto-induction media could not be found for *E. coli* BL21 (DE3) without plasmid (Additional file 1). In those cultivations, the shape of the OTR curves in the different media was in principle equivalent, showing a long phase of oxygen limitation interrupted by a diauxic break at about 8 h. Therefore, the impact of metabolic burden could be confirmed for the cultivations in Figure 4.

To analyze the OTR curves in more detail and to categorize the different OTR curves, two patterns of respiration behavior were distinguished. Both could be found for each *E. coli* variants tested. The first pattern (Merck, Roth_2, and OnEx_2) showed an exponential increase in OTR until a plateau of 35 to 60 mmol/L/h was reached for 2 to 3 h. This phase was followed by a second increase after 6 to 7 h cultivation time. In all cases a maximum OTR of around 70 mmol/L/h was reached. After 9 to 12 h, a sharp drop in OTR indicated the depletion of the initial carbon sources. The second pattern (AppliChem, DSM, Difco, Roth_1, Roth_4, and OnEx_1) revealed an exponential increase until the maximum oxygen transfer capacity of the system (approx. 70 mmol/L/h) was reached due to oxygen limitation [38]. Afterwards, the OTR either remained constant until all carbon sources were depleted or showed a short drop followed by an increase back to the maximum OTR. Medium prepared with Roth_3 yeast extract, however, could not clearly be assigned to one of the two described patterns of respiration behavior. The course of the OTR was comparable to the second pattern but the first increase in OTR already stopped at just 60 mmol/L/h, in line with the behavior of the first pattern.

Since auto-induction media lead to parallel induction of all cultivations at the same biomass concentration [32], the effects of changing yeast extract lots or varying commercial auto-induction medium lots on metabolic activity in induced cultures could easily be compared. Both, lots from different suppliers and lots from the same supplier (Roth, Germany) exhibited clear variations in respiration behavior. These observations reveal that chemical lot-to-lot variations can clearly be visualized by applying the RAMOS technique. Due to the fact that the reproducibility of all OTR curves was excellent, phenotyping of new lots or discrimination of different lots can easily be realized by only one shake flask experiment in the RAMOS device.

To correlate respiration activity with functional enzyme expression, volumetric activity of the two produced enzymes HBD and GDH was measured at the end of the cultivation (Figure 4C, F, and I). The qualitative trend to produce either high or low amounts of recombinant protein in TB+lactose medium with different yeast extract lots was comparable for both *E. coli* variants tested (Figure 4C and F). Poor enzyme expression was reached in TB+lactose medium prepared with DSM, Roth_1, Roth_3, and Roth_4 yeast extract (0.1 U/mL for HBD and less than 0.5 U/mL for GDH). Highest recombinant protein production could be observed in the medium prepared with AppliChem yeast extract (0.3 U/mL for HBD and 1.5 U/mL for GDH). The best performing Roth yeast extract could be identified to be Roth_2 with a nearly twofold increase in volumetric activity compared to the other Roth yeast extract lots tested.

The results of volumetric activity in TB+lactose medium clearly confirmed the observed differences in respiration behavior. Several groups [6,8,13,32] already observed that the raw material source and fabrication of yeast extract was responsible for the level of protein expression irrespective of the expression host. Grossmann et al. found cAMP, which is involved in high-level transcription of lac genes [39,40], to stimulate leaky expression in systems where the T7 RNA polymerase gene is expressed under control of lac operon elements [41]. Baracat-Pereira et al. assumed that precursors or inducers of cAMP could be present in yeast extracts working as transcription enhancer [13]. Therefore, varying cAMP concentrations in yeast extracts might be one explanation for the tremendous differences in recombinant protein production observed in the experiments in Figure 4.

It still remains unclear if only the ability to promote leaky expression is responsible for the enormous differences in respiration behavior and product formation. Since no significant variations in respiration behavior could be observed in TB medium under non-induced conditions (Figure 2), the cAMP availability might not cause the variations under induced conditions. For the production of yeast extract, yeast is usually grown on undefined media containing molasses. This complex ingredient might vary in its chemical composition resulting in variations of the yeast quality. Also differences in the yeasts’ autolysis process may contribute to the observed different metabolic behavior of the investigated strains. Therefore, it might also be possible that another ingredient in the yeast extract or the particular chemical composition of yeast extracts was responsible for a more efficient protein expression towards active and functional enzymes of some of the lots, as it has already been published for other organisms [6,12]. Hence, especially the ratio of specific amino acids in yeast extracts could possibly enhance or reduce the rate of recombinant protein synthesis. Ramirez et al. reported that addition of a specific amino acid could, on the one hand, lead to higher recombinant protein expression if the produced protein had a high content of the added amino acid. On the other hand, the added amino acid could repress biosynthetic pathways of other amino acids or metabolites leading to diminished yields of foreign proteins [15]. Since the composition of the yeast extracts was not a focus of this work, the dependency of functional protein production on certain ingredients was not investigated further.

In Figure 4I, the volumetric activities after cultivation in the two commercial OnEx medium lots are depicted. It should be noted that both lots were purchased from the same manufacturer. The two lots revealed not only large differences in respiration behavior but also in volumetric activity. After 14 h of cultivation, the most significant difference could be observed in GDH activity. OnEx_2 exhibited a fourfold higher activity for GDH compared to OnEx_1. In case of the enzyme HBD, the activity in OnEx_1 medium was 1.3-fold higher than in OnEx_2 medium.

The comparison of the volumetric activities in commercial OnEx auto-induction medium and self-made TB+lactose medium revealed the higher efficiency of the commercial medium in promoting functional recombinant protein expression in *E. coli*. Only TB+lactose medium prepared with AppliChem yeast extract showed activities comparable to those obtained with OnEx_2 medium for HBD and OnEx_1 medium for GDH. Since the detailed composition of OnEx medium is not published by the manufacturer, a comparison of the commercial and self-made medium is not feasible for absolute values of volumetric activity. As already demonstrated in different studies, an increased availability of yeast extract in cultivation medium can enhance the yield of recombinant protein [8,42-44]. Therefore, differences in the composition of self-made and commercial medium would preclude a comparison of product yield.

The medium lots pooled into pattern I and pattern II of respiration behavior (Figure 4) did not reveal a clear trend to produce either high or low amounts of functional recombinant protein. Low volumetric enzyme activities could be determined for medium lots of pattern II respiration behavior, except for two lots with high enzyme activities. Medium lots belonging to pattern I of respiration behavior exhibited medium to high values for the measured enzyme activity. However, the measurements of enzyme activity might not exhibit the highest possible volumetric activities reached in the course of cultivation. The volumetric activity was measured as conventional endpoint analysis in these experiments. Hence, the degradation of the produced enzymes might have started before the experiment had been terminated. A complete picture of recombinant protein production can only be obtained by offline analysis over the course of fermentation. Nevertheless, the lot-to-lot variations were clearly visualized by online measurement of respiration activity of *E. coli* in the performed cultivations and could be confirmed by huge differences in volumetric enzyme activity.

For heterologous protein production in *E. coli*, variations in respiration behavior and volumetric activity were expected for the two different auto-induction media (TB+lactose medium and OnEx medium) as well as the yeast extracts of different suppliers (Table 1). Fu et al. already observed different yields of a fusion protein when the *E. coli* strain was cultivated in complex medium with yeast extracts of different manufacturers [8]. However, it was remarkable that yeast extracts of the same supplier and also the commercial OnEx medium exhibited large lot-to-lot variations. These findings demonstrate that the production of recombinant proteins in cultivation media supplemented with complex components may be prone to significant variations if the quality of the raw material for medium preparation is not thoroughly checked.

**Figure 5:**
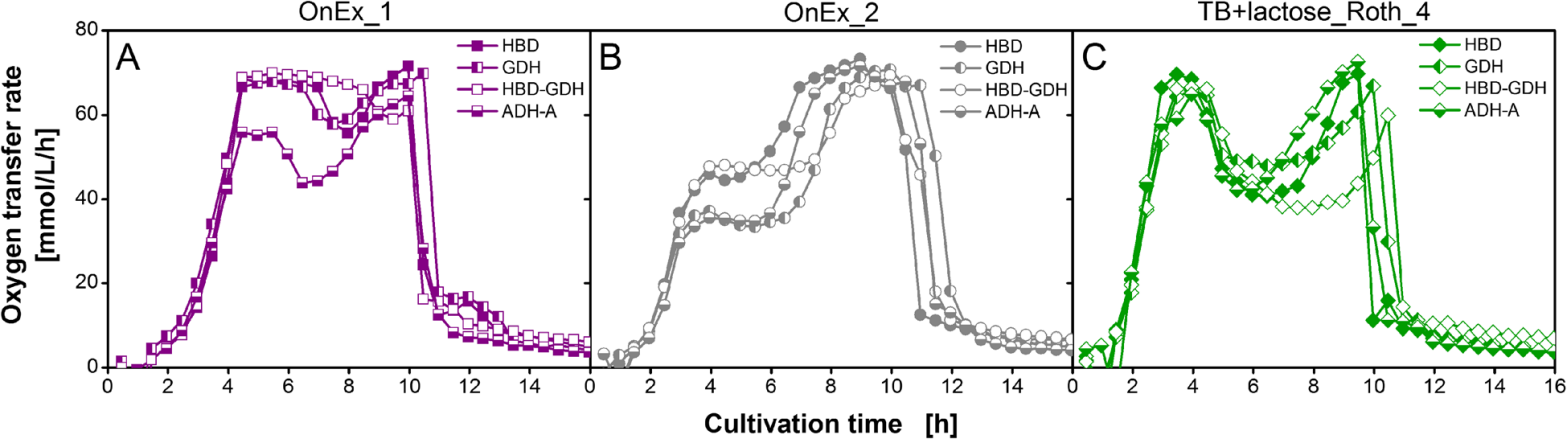
**Comparison of oxygen transfer rates of all** ***E. coli*** **variants in selected auto-induction media.** Oxygen transfer rate during cultivation of *E. coli* BL21 (DE3) variants expressing different recombinant target proteins (3-hydroxybutyryl-CoA dehydrogenase (HBD), glucose 1-dehydrogenase (GDH), HBD-GDH, and alcohol dehydrogenase A (ADH-A)) in Overnight Express auto-induction medium (**(A)** OnEx_1 and **(B)** OnEx_2) and **(C)** auto-inducing TB+lactose medium (Roth_4). The respiration behavior is categorized into two patterns, as described in the caption of Figure 4. Conditions: 250-mL flask, filling volume 10 mL, shaking frequency 350 rpm, shaking diameter 50 mm, and 37°C.

To further confirm the impact of complex medium components on other *E. coli* expression systems, the trend of respiration behavior as function of time is compared for all recombinant *E. coli* variants (listed in Figure 1) in three different auto-induction media in Figure 5. It needs to be added that the ADH-A is mainly produced in inclusion bodies under the investigated cultivation conditions [45]. In Figure 5A, OTR curves in OnEx_1 medium are depicted. Besides *E. coli* ADH-A, the respiration behavior of all variants could be assigned to the second pattern, since the OTR was exponentially increasing until the maximum oxygen transfer capacity of the system was reached. *E. coli* ADH-A showed essentially the same respiration behavior except for the first OTR increase to only 55 mmol/L/h. Figure 5B depicts the results of cultivation in OnEx_2 medium. The OTR curves of all variants clearly correlate to the first pattern of respiration behavior having a first OTR plateau between 35 to 50 mmol/L/h followed by a second increase to approx. 70 mmol/L/h. In Figure 5C, the respiration behavior is shown for TB+lactose medium prepared with Roth_4 yeast extract. All OTR curves exhibited respiration behavior belonging to the second pattern.

Although differences in respiration behavior between the four recombinant *E. coli* variants became apparent in Figure 5, the general trend of the OTR curves in the three examined media was comparable. Hence, it can be assumed that at induced conditions the respiration activity of the *E. coli* variants investigated in this study is linked to the complex medium lots used and to a much lesser extent to the specific strain. Furthermore, the impact of different complex medium lots on protein expression is visible in the respiration activity for *E. coli* variants producing soluble recombinant proteins (HBD, GDH) as well as proteins in inclusion bodies (ADH-A).

**Figure 6:**
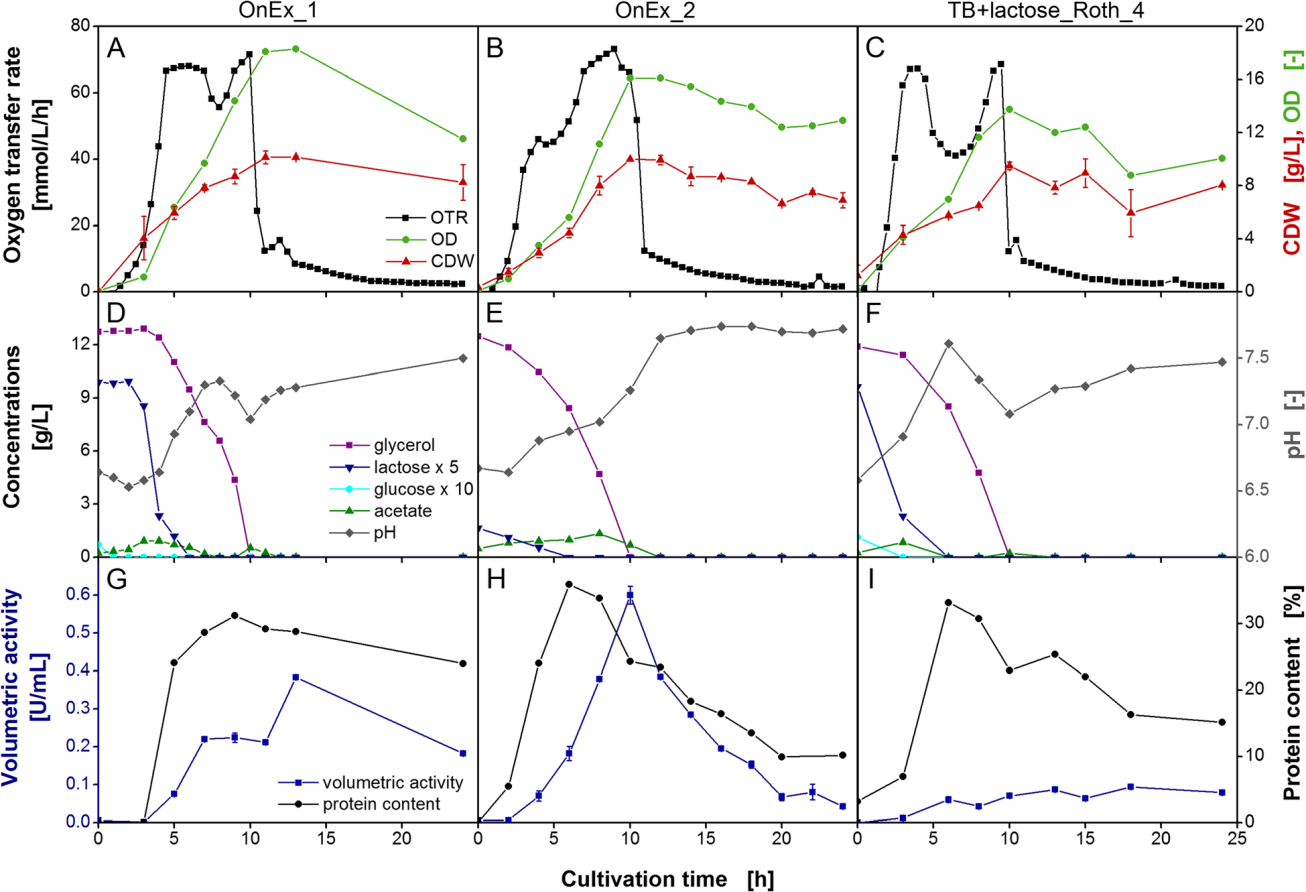
**Detailed characterization of selected auto-induction media with** ***E. coli*** **HBD.** Characteristic growth parameters of *E. coli* BL21 (DE3) expressing 3-hydroxybutyryl-CoA dehydrogenase (HBD) in Overnight Express auto-induction medium (OnEx_1 and OnEx_2) and in auto-inducing TB+lactose medium (Roth_4). Conditions: 250-mL flask, filling volume 10 mL, shaking frequency 350 rpm, shaking diameter 50 mm, and 37°C. **(A-C)** Oxygen transfer rate (OTR), cell dry weight (CDW), and optical densitiy (OD). The respiration behavior is categorized into two patterns, as described in the caption of Figure 4. **(D-F)** pH-value and glycerol, lactose, glucose, and acetate concentrations. To improve data visualization, lactose concentration was multiplied with a factor of 5 and glucose concentration with a factor of 10, respectively. **(G-I)** Volumetric activity and protein content of recombinant protein per total protein.

For an in-depth characterization of cultivation and enzyme expression in *E. coli*, additional RAMOS experiments were conducted in OnEx_1 medium, OnEx_2 medium, and TB+lactose medium with Roth_4 yeast extract. *E. coli* HBD was examined as representative of the four *E. coli* variants. The results for two other variants (*E. coli* GDH and *E. coli* ADH-A) are included as Additional files 2 and 3 in the appendix. Offline analysis was performed measuring biomass formation, carbon source consumption, acetate formation, pH, and expression of recombinant protein in conventional shake flasks under the same conditions. The results are presented in Figure 6.

*E. coli* HBD cultivated in OnEx_1 medium started to grow on the carbon source glucose (Figure 6D). After the depletion of glucose after 2 h, glycerol and lactose were consumed in parallel [32]. After 5 h, the culture entered a phase of oxygen limitation which is characterized by a constant OTR and a linear increase in the OD (Figure 6A). Lactose was depleted after about 7 h, indicated by a short drop in OTR. After 10 h, glycerol was also fully consumed and the OTR started to decrease. At that time, the highest biomass concentration could be reached with an optical density of 18 and a cell dry weight of 9 g/L. The changing pH profile (Figure 6D) during the cultivation could be assigned to acetate production and depletion and also to consumption of complex media components [22]. Acetate was formed as overflow metabolite during the cultivation.

In Figure 6G, the profile of volumetric HBD activity and the target protein content in the biomass – meaning the ratio of recombinant protein produced per total *E. coli* protein – is presented. Since glucose is known to repress the uptake of the inducer lactose into the cells [39], no recombinant protein was produced as long as glucose was present in the cultivation medium. Consequently, protein content and volumetric activity started to increase after glucose was fully consumed. Up to the point when lactose was depleted, the cells exhibited the highest target protein content in the cultivation reaching 30% of total *E. coli* protein. Volumetric activity was constant between 7 and 11 h and increased by the end of the cultivation from 0.2 U/mL to 0.4 U/mL. In additional experiments (data not shown), the peak in HBD activity was also observed after 13 to 14 h which correlated with the final decrease in OTR after all carbon sources were depleted.

In contrast to the cultivation of *E. coli* HBD in OnEx_1 medium, OnEx_2 medium exhibited a respiration behavior classified as pattern I respiration (Figure 6B). The OTR started to increase exponentially and reached the first plateau at 40 mmol/L/h after 4 h. The constant OTR correlated with a linear increase in OD and CDW. After 5.5 h, the second exponential increase in OTR to 70 mmol/L/h was accompanied by an exponential increase in OD and CDW. Highest biomass concentration was reached after 10 h with an OD of 16 and a CDW of 9 g/L. Comparing the concentrations of glucose and lactose in the two lots of OnEx medium, it became apparent that OnEx_2 medium (Figure 6E) showed lower contents of lactose and glucose than OnEx_1 medium (Figure 6D), although the manufacturer denied a change in OnEx medium composition. Glucose was not detectable by HPLC analysis and lactose concentration was only a quarter of the concentration measured in OnEx_1 medium. Therefore, the parallel consumption of glycerol and lactose started at the beginning of the cultivation under formation of small amounts of acetate as overflow metabolite. Acetate was consumed after depletion of both initial carbon sources which was also visible in the profile of the pH (Figure 6E). Protein content of HBD per total cell protein started to increase shortly after the start of cultivation (Figure 6H). The peak value was reached at the end of the first OTR plateau and simultaneously with depletion of lactose after 5 h. A maximum volumetric activity of 0.6 U/mL could be measured after 10 h of cultivation, concurrent with the depletion of the carbon source glycerol. After reaching the peak values of target protein content and volumetric activity, respectively, both values decreased significantly. The differences in the time of highest target protein content and highest volumetric activity arose from the fact that recombinant protein production per cell depends on the presence of the inducer lactose in the cultivation medium. After the peak value of target protein content, the ratio of expressed active enzyme per produced biomass shifted due to the fact that the inducer was depleted in the medium resulting in overgrowth of productive cells by unproductive cells [32].

In TB+lactose medium prepared with Roth_4 yeast extract (Figure 6C), OTR of *E. coli* HBD was exponential for 4 h followed by a drop in OTR from 70 mmol/L/h to 40 mmol/L/h. After 10 h, the OTR reached its highest value and subsequently decreased sharply. At that time the highest biomass concentrations could be measured with an OD of 13 and a CDW of 9 g/L. Glucose was consumed within the first 3 h of cultivation (Figure 6F). Depletion of lactose could be attributed to the first decrease in OTR and the depletion of glycerol to the second OTR decrease, respectively. The peak value of 30% target protein content was reached after depletion of lactose followed by a subsequent decrease (Figure 6I). In contrast, volumetric activity did not exceed 0.1 U/mL and, therefore, reached only one quarter of activity in OnEx_1 medium and even only one sixth of activity in OnEx_2 medium.

Comparison of the commercial OnEx medium lots revealed that highest volumetric activities were obtained in different phases of cultivation. In OnEx_1 medium, the activity was highest at the beginning of stationary phase (Figure 6G). In contrast, OnEx_2 medium exhibited highest activity after depletion of glycerol instantly followed by a sharp decrease (Figure 6H). Furthermore, the peak value was 1.5-fold higher in OnEx_2 medium compared to OnEx_1 medium. This observation shows that the measurement of enzyme activity at the end of a cultivation in different media, as it was done in Figure 4, does not automatically display the best performing cultivation. For a screening of different yeast extracts or other complex medium ingredients, it might be preferable to measure the activity in a distinct cultivation phase, for instance with depletion of glycerol. Thereby, the misleading influence of possible degradation of the recombinant protein could be avoided. Furthermore, glycerol depletion is clearly indicated in respiration behavior by the last strong decline in OTR and, therefore, RAMOS cultivations can be used to trigger taking samples in exactly the same phase of cultivation.

The fact that no glucose and lower amounts of lactose were detectable in the commercial OnEx_2 medium (Figure 6E) could potentially explain the differences in volumetric activity. Blommel et al. reported that enzyme expression in auto-induction medium was dependent on the oxygenation state of the culture [31]. Protein yield was much lower when cultivations were conducted under oxygen unlimited conditions. Interestingly, much higher yields could be obtained by decreasing the glucose concentration. Studier observed the same effect [32]. However, lowering the concentration of the inducer lactose had the opposite effect. Therefore, we can assume that the lower amounts of glucose and of the inducer lactose in OnEx_2 medium cannot be the only reason for higher protein production. The variation in carbon source availability in the two commercial medium lots can also not explain the differences in respiration behavior. TB+lactose medium prepared with Merck or Roth_2 yeast extract showed the same respiration behavior as OnEx_2 medium (Figure 4). The same glucose and lactose concentrations as in OnEx_1 medium were added to these media. Hence, the deviating respiration behaviors and enzyme activities of OnEx_1 and OnEx_2 medium could be considered to result from the lot-to-lot variation of complex medium components.

In TB+lactose medium prepared with Roth_4 yeast extract, only basal levels of volumetric HBD activity were detectable. Due to the discrepancy between activity and protein content, it can be assumed that the majority of expressed recombinant enzymes accumulated in inclusion bodies in a non-active form. This has to be verified in future investigations. The low ratio of volumetric activity to expressed protein might result from a differed nutrient composition of the medium lot and, therefore, yeast extract lot used. Sorensen and Mortensen already reported that medium composition can have a big impact on soluble expression of recombinant protein [46]. Hence, lot-to-lot variations in yeast extracts might not only determine the protein expression rate in *E. coli*, but also the tendency to express functional recombinant proteins.

A variation in complex component lot or yeast extract lot appears to also be the reason for the different levels of protein degradation at the end of the cultivation. In OnEx_2 medium and TB+lactose medium, a clear trend of protein degradation was visible in the stationary cultivation phase (Figure 6H and I). In OnEx_1 medium, the target protein content remained almost unchanged. To prove this assumption, also different yeast extract lots need to be tested in cultivations with parallel offline analysis.

## Conclusions

In this study, the Respiration Activity Monitoring System (RAMOS) was used to evaluate the impact of different yeast extracts and commercial Overnight Express (OnEx) medium lots on metabolic activity and product yield of recombinant *E. coli* variants encoding different enzymes. Under non-induced conditions, respiration activity of *E. coli* was not affected by a variation of the supplemented yeast extract lot. Cultivations in auto-induction media with exactly the same yeast extract lots or complex component lots led to reproducible and reliable results indicating the applicability of RAMOS for this evaluation. The comparison of *E. coli* cultivations under induced conditions exhibited tremendous differences in respiration behavior and volumetric activity for the two investigated auto-induction media (self-made and commercial) as well as for yeast extract lots of different suppliers. Moreover, distinct lot-to-lot variations could also be detected for yeast extracts of the same supplier and the commercial OnEx auto-induction medium lots.

The cultivations with the two different commercial OnEx auto-induction medium lots exhibited the highest volumetric activity at different cultivation times, probably due to differences in respiration behavior. Only by online monitoring of the cultures, a distinct cultivation phase (e.g. glycerol depletion) can be detected and chosen for comparable and reproducible offline analysis of the yield of functional product.

These findings demonstrate that cultivations conducted in complex media may be prone to significant variation in final product quality and quantity if the quality of the raw material for medium preparation is not thoroughly checked. In the PAT initiative, the control of raw materials is one of the key aspects for biopharmaceutical production processes. However, a conventional biochemical analysis of complex raw materials might not be sufficient to maintain process consistency. Complex component lots always exhibit small differences in the concentration of specific ingredients. Although, these variances may be within the predefined tolerance level, the risk to affect final product quality in a specific production process persists. In this study, the RAMOS technique enabled a screening and phenotyping of complex raw material lots by online measurement of the respiration activity. Consequently, the quality of complex raw material lots can efficiently be assessed if the distinct effects on culture behavior and final product quality and quantity are visualized. In future investigations, this method should also be tested for proteins that are produced via constitutive expression and IPTG induced expression to confirm the universal applicability. In addition, it remains to be examined if the *E. coli* system investigated in this work can also serve as a bioassay for different biological systems applying yeast extract.

## Methods

### Microorganisms

All experiments were performed with recombinant *Escherichia coli* BL21 (DE3) (Novagen, Merck, Germany) containing different plasmids. The plasmid pET11a (ampicillin resistant) encoded the 3-hydroxybutyryl-CoA dehydrogenase (HBD, TTHA1262) from *Thermus thermophilus* HB8 with a molecular weight of 32 kDa. This plasmid was taken from the *Thermus thermophilus* HB8 gene expression library obtained from RIKEN BioResource Center, Japan [47]. The plasmid pACYCDuet-1 (chloramphenicol resistant) encoded the glucose 1-dehydrogenase (GDH, SSO3003) from *Sulfolobus solfataricus* [48,49], a homotetramer with a molecular weight of 41 kDa per subunit. The plasmid pET22b (ampicillin resistant) encoded the alcohol dehydrogenase A (ADH-A) from *Rhodococcus ruber* DSM 44541 [50], a homotetramer with a molecular weight of 35 kDa per subunit. All plasmids were obtained from Novagen, Merck, Germany.

In total, four *E. coli* variants were examined in the experiments: *E. coli* pET11a-hbd (*E. coli* HBD), *E. coli* pACYCDuet-1-dhg-1 (*E. coli* GDH), *E. coli* pET22b-adh-a (*E. coli* ADH-A), and *E. coli* pET11a-hbd-pACYCDuet-1-dhg-1 (*E. coli* HBD-GDH). In the latter case, *E. coli* was transformed with two plasmids encoding HBD and GDH. Stock cultures of all variants were prepared by cultivation in TB medium. Cells were harvested in the exponential growth phase and stored in 1 mL vials with a final glycerol concentration of 150 g/L at −80°C.

### Media and solutions

Under non-induced conditions, *E. coli* BL21 (DE3) was cultivated in terrific broth (TB) medium with 5 g/L glycerol. The medium consists of 12 g/L tryptone, 12.54 g/L K_2_HPO_4_, 2.31 g/L KH_2_PO_4_ (all ingredients from Roth, Germany), and 24 g/L yeast extract (different suppliers, see Table 1) dissolved in water. The pH-value was 7.2 ± 0.2 without adjustment. For growth under induced conditions, two different types of complex auto-induction media were used. The commercially available Overnight Express (OnEx) auto-induction medium (Novagen, Merck, Germany) is prepared from 60 g/L OnEx medium granulate and 12.6 g/L glycerol dissolved in water without pH adjustment. The TB+lactose medium contains TB medium with 12.6 g/L glycerol, 2 g/L lactose, and 0.5 g/L glucose as carbon sources. The pH was adjusted with 10% (v/v) HCl to a value of 6.78 to be comparable to the OnEx medium. After autoclaving, 0.1 g/L ampicillin and/or 0.034 g/L chloramphenicol were added to the medium under sterile conditions depending on the selection markers of the plasmids used. Table 1 gives an overview of the yeast extract lots (used for preparation of TB medium and TB+lactose medium) and the OnEx medium lots and discloses the nomenclature for the experiments.

### Cultivation

All cultivations were performed in 250-mL shake flasks with a filling volume of 10 mL. The cultures were incubated at 37°C using an orbital shaker (LT-X (Lab-Therm), Kühner, Germany) with a shaking diameter of 50 mm and a shaking frequency of 350 rpm. Precultures were made for all cultivations. For precultivation, TB medium prepared with Roth_4 yeast extract was inoculated with 200 μL stock culture of the desired variant and cultivated under the before-mentioned conditions. Cells were harvested in the exponential phase after 3 h to prevent a possible impact on the main culture due to leaky expression in the stationary phase [41] or saturation of the culture with ß-lactamase going along with plasmid loss [51]. Main cultures were inoculated with the precultures to a final optical density (OD_600_) of 0.1.

### Respiration Activity Monitoring System (RAMOS)

In all main cultures, the respiration activity of the organisms was monitored by an in-house manufactured Respiration Activity Monitoring System (RAMOS) determining the oxygen transfer rate (OTR) [17,18]. Conventional shake flasks are equipped with a gas inlet and outlet and an oxygen sensor measuring the oxygen partial pressure in the headspace of the flasks. During the cultivation, a measuring cycle is continuously repeated consisting of a rinsing phase and a measuring phase. During rinsing phase, the air flow is adjusted in a way that the gas concentration in the headspace of the modified shake flasks is equal to that in conventional shake flasks sealed with cotton plugs [52]. During measuring phase, the gas inlet and outlet valves are closed and the respiration activity of the organisms leads to a decrease in oxygen partial pressure. From this change in oxygen partial pressure, the OTR can be calculated. Commercial versions of RAMOS can be purchased from Kühner AG, Birsfelden, Switzerland or HiTec Zang GmbH, Herzogenrath, Germany.

### Sample analytics

For offline analysis, samples were taken from conventional shake flasks sealed with cotton plugs and cultivated in parallel under the same conditions as the RAMOS flasks. Cell density was quantified by measurement of optical density at a wavelength of 600 nm (OD_600_) in 1-cm cuvettes in a photometer (Genesys 20, Thermo Scientific, Germany) in duplicates. Samples were diluted with fresh medium to keep OD_600_ in the linear range between 0.1 and 0.5. Fresh medium was used as blank. Cell dry weight (CDW) was gravimetrically determined from cell pellet of 1 mL culture broth in duplicates. The pH-value of the culture broth was measured with a CyberScan pH 510 (Eutech Instruments, The Netherlands).

Concentrations of the carbon sources glucose, lactose, glycerol, and acetate were determined by HPLC (Ultimate 3000, Dionex, USA) equipped with an Organic Acid-Resin-Column (250 × 8 mm, CS-Chromatographie Service, Germany) and an Organic Acid-Resin-Precolumn (40 × 8 mm, CS-Chromatographie Service, Germany). The column was eluted with 5 mM H_2_SO_4_ at 60°C and 0.8 mL/min flow rate. Peaks were detected with a Shodex RI-101 refractometer (Showa Denko Europe, Germany). Data analysis was performed with the software Chromeleon 6.2 (Dionex, Germany).

Recombinant protein production was investigated by sodium dodecylsulfate polyacrylamide gel electrophoresis (SDS-PAGE). Cell pellet was diluted with NuPAGE® LDS Sample Buffer (Invitrogen, Germany) to OD_600_ = 5 and heated to 70°C for 10 min. NuPAGE® Bis-Tris Gels (4–12%, Invitrogen, Germany) were mounted in a XCell SureLock® Mini-Cell (Invitrogen, Germany). The chamber was filled up with NuPAGE® MES SDS Running buffer (Invitrogen, Germany) and gels were loaded with 20 μL of prepared samples and 10 μL protein standard (Roti®-Mark Standard, Roth, Germany). After running the gels at 200 V and 120 mA for 35 min, gels were stained overnight with Roti®-Blue (Roth, Germany) at room temperature.

Analysis of the SDS gels by densitometry was done within the software TotalLab TL100 (Nonlinear Dynamics, UK) using one-dimensional gel analysis: lanes were created automatically; background was subtracted using the rolling ball method with a radius of 100; and detection of protein bands was done with a minimal slope of 100. The resulting percentage of target protein’s band determines the fraction of recombinant protein per total protein of the cells (protein content, given in percent).

Volumetric activities of produced 3-hydroxybutyryl-CoA dehydrogenase (HBD) and glucose 1-dehydrogenase (GDH) were determined at 70°C by following either the oxidation of NADH or the reduction of NAD^+^ at a wavelength of 340 nm using a Uvikon 922A spectrophotometer (Kontron Instruments, Italy) equipped with a temperature-controlled cuvette holder. Enzyme solutions containing HBD and/or GDH were prepared from *E. coli* cell pellet suspended in 0.5 M Tris buffer (pH 8, RT) by heating for 15 min at 70°C. The heat treated cells were centrifuged for 20 min at 14.000 rpm obtaining the supernatant (enzyme solution). The reaction mixture for measurement of HBD activity contained appropriate amounts of enzyme solution (around 10% v/v), 100 mM Tris buffer (pH 8, RT), 100 mM 2,5-hexanedione, and 0.2 mM NADH. The reaction mixture for measurement of GDH activity contained appropriate amounts of enzyme solution (around 2% v/v), 100 mM Tris buffer (pH 8, RT), 200 mM glucose, and 1 mM NAD^+^. In all assays, the reactions were started by addition of respective cofactors. One unit (U) is defined as the amount of enzyme converting 1 μmol cofactor per min. Temperature-dependent degradation of NADH was corrected for.

The volumetric activity of produced alcohol dehydrogenase A (ADH-A) was determined at 30°C by following the oxidation of NADH at a wavelength of 340 nm in 96-well microtiter plates (F-profile, Roth, Germany) using a Synergy-4 Multi-Mode Microplate Reader (BioTek Instruments, Germany). To disrupt cells of *E. coli* ADH-A, cell pellet of 5 mL culture broth was suspended in 1 mL BugBuster Protein Extraction Reagent (Novagen, Merck, Germany) adding 1000 U/mL lysozyme (Roth, Germany) and 25 U/mL DNaseI (AppliChem, Germany). Cell disruption was continued according to the manufacturers’ specifications obtaining the soluble fraction with dissolved ADH-A. 200 μL reaction mixture were prepared for measurement of ADH-A activity and contained 50 mM Tris buffer (pH 8, RT), 100 mM 2,5-hexanedione, and 0.5 mM NADH. The reactions were initiated by addition of appropriate amounts of enzyme solution (around 0.02% v/v). One unit (U) is defined as the amount of enzyme converting 1 μmol cofactor per min.

## Additional files

Additional file 1:
**Cultivation of** ***E. coli*** **without plasmid in auto-induction media.** Oxygen transfer rate during cultivation of *E. coli* BL21 (DE3) without plasmid in self-made auto-inducing TB+lactose medium with various yeast extracts as listed in Table 1 and commercial Overnight Express (OnEx_2) auto-induction medium. Conditions: 250-mL flask, filling volume 10 mL, shaking frequency 350 rpm, shaking diameter 50 mm, and 37°C. Additional file 2:
**Detailed characterization of selected auto-induction media with** ***E. coli*** **GDH.** Characteristic growth parameters of *E. coli* BL21 (DE3) expressing glucose 1-dehydrogenase (GDH) in Overnight Express auto-induction medium (OnEx_1 and OnEx_2) and in auto-inducing TB+lactose medium (Roth_4). Conditions: 250-mL flask, filling volume 10 mL, shaking frequency 350 rpm, shaking diameter 50 mm, and 37°C. (A-C) Oxygen transfer rate (OTR), cell dry weight (CDW), and optical densitiy (OD).(D-F) pH-value and glycerol, lactose, glucose, and acetate concentrations. To improve data visualization, lactose concentration was multiplied with a factor of 5 and glucose concentration with a factor of 10, respectively. Dotted lines visualize the exact time of glycerol depletion. (G-I) Volumetric activity and protein content of recombinant protein per total protein. Additional file 3:
**Detailed characterization of selected auto-induction media with** ***E. coli*** **ADH-A.** Characteristic growth parameters of *E. coli* BL21 (DE3) expressing alcohol dehydrogenase A (ADH-A) in Overnight Express auto-induction medium (OnEx_1 and OnEx_2) and in auto-inducing TB+lactose medium (Roth_4). Conditions: 250-mL flask, filling volume 10 mL, shaking frequency 350 rpm, shaking diameter 50 mm, and 37°C. (A-C) Oxygen transfer rate (OTR), cell dry weight (CDW), and optical densitiy (OD). (D-F) pH-value and glycerol, lactose, glucose, and acetate concentrations. To improve data visualization, lactose concentration was multiplied with a factor of 5 and glucose concentration with a factor of 10, respectively. Dotted lines visualize the exact time of glycerol depletion and the initial concentration of lactose in OnEx_1 medium. (G-I) Volumetric activity and protein content of recombinant protein per total protein.

## Abbreviations

FDA: Food and Drug Administration
PAT: Process Analytical Technology
*E. coli*: *Escherichia coli*
LB medium: Lysogeny broth medium
TB medium: Terrific broth medium
RAMOS: Respiration Activity Monitoring System
OTR: Oxygen transfer rate
HBD: 3-hydroxybutyryl-CoA dehydrogenase
GDH: Glucose 1-dehydrogenase
ADH-A: Alcohol dehydrogenase A
OnEx medium: Overnight Express auto-induction medium
CDW: Cell dry weight
OD: Optical density
